# Valproic acid decreases urothelial cancer cell proliferation and induces thrombospondin-1 expression

**DOI:** 10.1186/1471-2490-12-21

**Published:** 2012-08-16

**Authors:** Timothy K Byler, Dean Leocadio, Oleg Shapiro, Gennady Bratslavsky, Christopher J Stodgell, Ronald W Wood, Edward M Messing, Jay E Reeder

**Affiliations:** 1 Department of Urology, State University of New York Upstate Medical University, 750 East Adams Street, Syracuse, NY, 13210, USA; 2 Department of Obstetrics and Gynecology, University of Rochester, 601 Elmwood Avenue, Rochester, NY, 14642, USA; 3 Department of Urology, University of Rochester, 601 Elmwood Avenue, Rochester, NY, 14642, USA

**Keywords:** Bladder cancer, Valproic acid, Thrombospondin-1, Urothelial carcinoma, Gene expression

## Abstract

**Background:**

Prevention of bladder cancer recurrence is a central challenge in the management of this highly prevalent disease. The histone deacetylase inhibitor valproic acid (sodium valproate) has anti-angiogenic properties and has been shown to decrease bladder cancer growth in model systems. We have previously shown reduced expression of thrombospondin-1 in a mouse model and in human bladder cancer relative to normal urothelium. We speculated that inhibition of angiogenesis by valproate might be mediated by this anti-angiogenic protein.

**Methods:**

Bladder cancer cell lines UMUC3 and T24 were treated with valproate or another histone deacetylase inhibitor, vorinostat, in culture for a period of three days. Proliferation was assessed by alamar blue reduction. Gene expression was evaluated by reverse transcription of RNA and quantitative PCR.

**Results:**

Proliferation assays showed treatment with valproate or vorinostat decreased proliferation in both cell lines. Histone deacetylase inhibition also increased relative expression of thrombospondin-1 up to 8 fold at 5 mM valproate.

**Conclusions:**

Histone deacetylase inhibitors warrant further study for the prevention or treatment of bladder cancer.

## Background

Bladder cancer is the fourth most commonly diagnosed cancer in the United States with over 60,000 new cases per year [1]. Fortunately, the majority of these cancers are superficial and successfully treated surgically. Unfortunately, these patients require intense follow-up due to high recurrence rates and the potential for progression to invasive cancer. Cystoscopy is recommended at regular intervals and even the lowest risk patients have a 30% recurrence rate at 5 years [2]. This constant need for surveillance imposes economic and life style hardship. An effective therapy to decrease bladder cancer recurrence could have significant impact on both quality of life and survival for over 500,000 patients with a history of bladder cancer in the United States alone.

Post-translational histone modifications such as acetylation are associated with transcriptionally active regions of the genome. Histone deacetylation appears to be a mechanism whereby cancers decrease expression of genes involved in cell cycle control and apoptosis. Histone deacetylase inhibitors (HDACi) are an emerging class of cancer drugs that might be useful in preventing bladder cancer recurrence. Valproic acid (sodium valproate) is a relatively weak HDACi but has demonstrated potential in the treatment of glioblastomas [3], thyroid cancer [4], and leukemia [5]. There are several on-going clinical trials of valproate for the treatment of other cancers registered on ClinicalTrials.gov. Extensve clinical experience with valproate as a seizure medication demonstrates that it is generally a well-tolerated drug that can be administered for long periods. For these reasons valproate is an attractive candidate for the prevention of bladder cancer recurrence.

Anti-neoplastic properties of valproate in bladder cancer models have recently been reported by several groups. Valproate decreased proliferation (or viability) of TCC-SUP, T24, RT4, and HT1376 cell lines; increased histone H3 acetylation and p21 expression and activated caspase 2 and caspase 3 in T24 cells [6]. In addition, in vitro invasiveness was decreased in valproate treated T24, TCC-SUP, and HT1376 cells. This is not restricted to in vitro studies: T24 xenografts had reduced growth with chronic administration of valproate (0.4% in drinking water for 35 days) in male athymic nu/nu mice. Similar results were reported by Byun et al. [7] for TCC-SUP and 5637 cell lines (identified in the publication by their ATCC Numbers as HTB5 and HTB9 respectively). Histone deacetylase 1 is expressed at higher levels in human bladder cancer compared to normal urothelium and its expression is also increased in the BBN mouse bladder cancer model [8]. These authors also reported delayed BBN-induced bladder tumors in mice. Valproate decreased proliferation in UMUC3, RT112, TCCSUP, and RT4 bladder cancer cell lines and, increased the percentage of cells in the G1 phase of the cell cycle with concomitant changes in cell cycle regulatory proteins [9].

Thrombospondin-1 (TSP1) is a well known natural inhibitor of angiogenesis. TSP1 anti-angiogenesis activity is mediated at least in part through the CD36 receptor, which initiates a cascade of events culminating in death of endothelial cells [10]. TSP1 expression in the urinary bladder is altered in bladder cancer and associated with low nuclear p53, increased tumor recurrence, and decreased survival [11]. Cultured bladder cancer cell lines stimulated to migrate and neovascularization showed lower TSP1 expression compared to normal urothelial cells, suggesting that bladder tumors may selectively down regulate TSP1 thus promoting angiogenesis [12].

We have previously shown that TSP1 expression is reduced in the bladders of UPII-SV40T transgenic mice relative to wildtype littermates [13]. UPII-SV40T mice develop bladder cancer due to urothelium-specific expression of the simian virus 40 T antigen protein [14]. Tumor growth was reduced and TSP1 expression increased by castration. One of us (CS) investigating the teratogenic properties of valproate noted that TSP1 expression was enhanced in embryos carried by dams treated with valproate (unpublished results). We speculated that the anti-angiogenic action of valproate might be due to increases in TSP1 expression in addition to a direct effect on cancer cell proliferation.

Here we report that valproate does induce TSP1 expression in bladder cancer cell lines and that this is likely mediated through HDAC inhibition. The latter was evidenced by increased TSP1 expression in response to another HDAC inhibitor vorinostat (SAHA).

## Methods

### Tissue culture

UMUC-3 (ATCC CRL-1749) and T-24 (ATCC HTB-4) bladder cancer cell lines were purchased from the American Type Culture Collection (ATCC). They were grown and subcultured in Dulbecco’s Minimal Essential Medium, 10% fetal bovine serum, and 1% penicillin/streptomycin media at 37C in a 5% CO_2_ incubator.

### HDAC inhibitors

Sodium valproate was purchased from Westward Pharmaceuticals (Eatontown, NJ) as a stock solution at 100 mg / ml. SAHA was purchased as a dry powder and reconstituted in dimethyl sulfoxide at 0.5 M and stored at -20C.

### Proliferation assay

Both cell lines were plated at low seed onto a 24 well plate. This was allowed overnight incubation. The following day, the media was removed and replaced with media containing preset concentrations of valproate or SAHA. These were incubated for 72 hours. At that point, the media was removed and media containing no treatment but supplemented with 10% Alamar blue was added. This was allowed to incubate for three hours at which point absorbance was read at 570 and 600 nm. Each condition had four replicates. The ratio of absorbance at 570 to 600 nm was scaled from zero for the no cell wells to 100% for the no treatment wells. The data were analyzed by t-test using JMP Statistical Software.

### Expression analysis

Cells were grown in 25 cm^2^ T-flasks and treated with valproate from 0 mM to 5 mM while SAHA was dosed at 1 μM and 5 μM. The cultures were viewed daily and ensured that the cells had not reached confluence. Cultures were carried out 72 hours at which time the cells were harvested for RNA extraction. This is comparable to previous reports in which a three-day incubation was needed prior to changes being evident [9]. Cells were photographed at day 0 and day 3 prior to RNA harvest.

### RNA extraction

After 72 hours treatment, the cells were scraped into PBS and RNA extracted using an RNAeasy kit (Qiagen). RNA was quantified using a NanoDrop spectrophotometer to measure absorbance at 260 nm. Yields ranged from 2.7 μg to 460 μg total RNA and were inversely proportional to HDAC inhibitor dose. The ratio of absorbance at 260 nm to absorbance at 280 was 2.0 to 2.1 for all specimens.

### Reverse transcription

Reverse transcription was performed according to manufacturer’s instructions using the Verso cDNA kit (Thermo) in a 20 μl reaction. One μg total RNA was denatured for 5 minutes at 70°C then cDNA synthesized for 30 minutes at 42°C utilizing random hexamer priming and the RNA enhancer additive.

### Quantitative PCR

Each cDNA reaction was diluted with 140 μL of molecular grade water. PCR primers all spanned at least one intron. Primer Details are in Table 1. The reactions consisted of 10 μL sybr green master mix (Roche), 1 μL of 5 mM primer each, and 8 μL of cDNA diluted template. PCR conditions were 95°C for 5 minutes, 95°C for 10 seconds, 60°C for 10 seconds, and 72°C for 30 seconds for 60 cycles. Melting analysis was performed from 65°C for to 97°C with 0.11 C/s ramp rate on a Roche Light Cycler 480. Primers included heat shock protein 90 (HSP 90), bax transmembrane protein (BAX), thrombospondin-1 (TSP1), ATP Synthase 5B, beta-actin and hemeoxygenase-1 (HO-1). Reference genes were selected according to Andersen [15]. All reactions were performed in triplicate.

**Table 1:** qPCR primers

**Target Gene**	**Primer 1**	**Primer 2**
ATP Synthase	GGCAGGGTCAGTCAAGTCAT	CCCTTCTGCTGTGGGCTAT
HSP 90	ATGCCTGAGGAAGTGCACCAT	CCAGACTTGGCAATGGTTCCC
BAX	TCAATATCAGGGAGCCCAAG	AGCAGCACCTGAAGAAGGTC
Beta-Actin	GTGGGCATGGGTCAGAAGGATTCC	GGCCAGAGGCGTACAGGGATAG
TSP-1	ACTGTCCATTCCATTACAACCCAGC	TGTCACACTGATCTCCAACCCCATCCA
HO-1	GGTGATAGAAGAGGCCAAGAC	GCAGAATCTTGCACTTTGTTG

### RT-PCR data analysis

A geometric mean was taken of the 4 reference genes and used a standard comparison [16]. The delta-delta CT method was used to calculate relative fold change in expression differences between samples [17]. The data were analyzed by t-test using JMP Statistical Software. Statistical significance was determined at the p < 0.05 level.

## Results

### Cell proliferation assay

**Figure 1:**
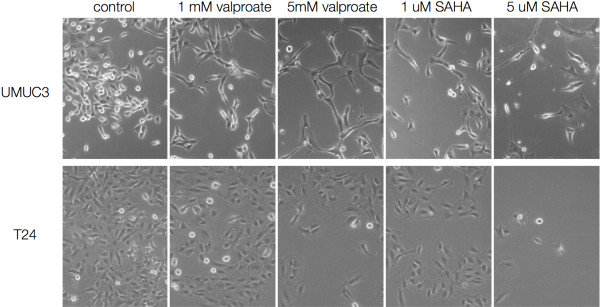
**Phase contrast images (original magnification 100X) showing the impact of HDAC inhibitor treatment at the indicated concentration for three days.** Note the lower cell numbers in the treated cells and morphological changes consistent with a more differentiated urothelial cell type.

**Figure 2:**
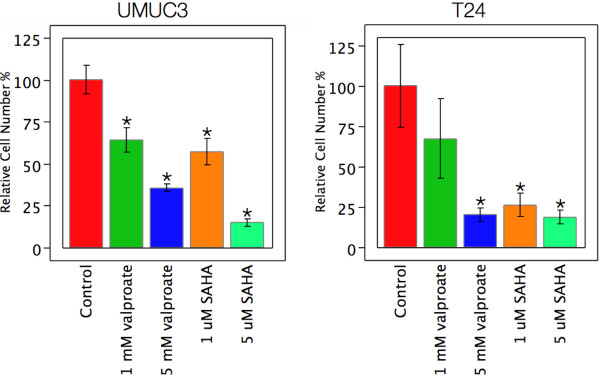
**Relative cell numbers by alamar blue assay for bladder cancer cell lines treated for three days with HDAC inhibitors at the indicated concentrations.** Statistically significant differences (t-test, p < 0.05) from control are indicated by *. There were statistically significant reductions in cell number for all treatment conditions relative to untreated controls for UMUC3 cells. Similarly, all treatments except the 1 mM valproate condition showed significant reduction in T24 cell number.

T24 and UMUC3 cell lines were treated with 1 mM and 5 mM valproate and 1 μM and 5 μM SAHA. Both cell lines showed a reduction in mitotic figures and proliferation under phase contrast (Figure 1). The UMUC3 cell line had a profound change in cellular morphology displaying long dendrite-like processes. Alamar blue was used to assay cell number following three days of drug exposure. Cell numbers were reduced by both drugs in both cell lines (Figure 2).

### TSP1 expression in response to HDAC inhibitors

**Figure 3:**
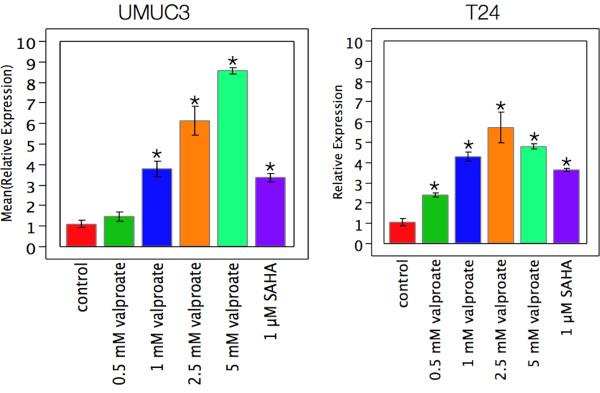
**Induction of TSP1 mRNA expression by HDAC inhibitors in bladder cancer cell lines.** Statistically significant differences (t-test, p < 0.05) relative to control are indicated by *. Cells were treated for three days with indicated drugs and concentrations. TSP1 expression increased with dose in both cell lines. The increase was statistically significant at and above 1 mM valproate for UMUC3 and at and above 0.5 mM valproate for T24 cells. SAHA treatment at 1 μM induced TSP1 in both cell lines but decreased cell viability at 5 μM to the point that RNA yields were insufficient for qPCR analysis.

TSP1 is an extracellular matrix protein whose expression was assessed using quantitative reverse transcription PCR (qPCR) and delta-delta-CT relative to the geometric mean of four reference genes, beta-actin, BAX, HSP90, and ATP Synthase. T24 and UMUC3 cells were grown in 25 cm^2^ tissue culture flasks and treated with 0.5, 1.0, 2.5, 5.0 mM valproate, and 1.0 or 5.0 μM SAHA for three days. At 5 μM SAHA RNA yields were insufficient for analysis indicating a cytotoxic dose. The qPCR results are presented in Figure 3. TSP1 expression in the UMUC3 cells was significantly increased at doses of 1.0 mM and higher and was over 8-fold higher relative to control at 5 mM. SAHA at 1 μM increased TSP1 expression more than three-fold as well. Similar results were obtained for the T24 cell line with a dose dependent increase in TSP1 expression, and was significant at 0.5 mM and higher concentrations of valproate reaching 6-fold levels at 5 mM. SAHA induced TSP1 expression almost four fold in the T24 cells.

## Discussion

The primary goal of our study was to investigate the effects of valproate on bladder cancer cells and provide a possible mechanism for these effects. First, we confirmed decreased proliferation with histone deacetylase inhibition in the two bladder cancer cell lines, T24 and UMUC-3. Second, we demonstrated that valproate increased TSP1 production, evidenced by increased mRNA expression. The UMUC-3 cell line also displayed profound morphological changes with valproate. The dendritic processes are consistent with urothelial umbrella cell differentiation. These data support the hypothesis that valproic acid exerts a negative effect on bladder cancer growth and shift to a more differentiated state.

TSP1 expression has been noted to be lower in bladder cancer specimens [13] and it is a potent anti-angiogenic mediator [18]. Other work suggests that valproate acid is an inhibitor of angiogenesis through direct effects on endothelial cells [19]. A connection between HDAC inhibition and TSP1 expression has not been reported. Our in-vitro work suggests that valproate acid may modify angiogenesis in cancer by its action on TSP1 expression. The exophytic growth of bladder tumors is dependent on angiogenic support, inhibition of angiogenesis could slow growth and possibly kill bladder tumors.

Valproate is a drug with a long clinical history for the treatment of seizures. The toxicity profile for valproate is acceptable for its possible use in chemoprevention of bladder cancer. The recommended therapeutic level of valproic acid for the treatment of seizures is generally accepted to be between 50–125 ug/mL in humans [20]. At the high end this serum level is 0.75 mM. A recent study looked at valproic acid induced proliferative changes in ovarian cancer cells [21] Cytotoxic effects of valproic acid were noted above 2.5 mM which is consistent with our findings.

Changes in RNA expression do not necessarily lead to changes in protein levels and we did not assess TSP1 protein levels in this in vitro study. TSP1 is a large multimeric secreted protein with biologically active cleavage products. Capture of the protein from media and/or the tissue culture substrate presents several technical challenges. In addition, it is not our contention that TSP1 acts on the cancer cell, rather that normalizing TSP1 expression in cancer cells could decrease angiogenesis through TSP1 action on endothelial cells.

HDAC inhibitors are attracting attention for the treatment of several cancers. For example, SAHA has been approved for the treatment of cutaneous T cell leukemia. Our data and previous reports show direct effects of both SAHA and valproate on bladder cancer cells *in vitro* and suggest that anti-angiogenic properties of this class of drugs could be mediated through induction of the anti-angiogenic protein TSP1. An effective low cost drug such as valproate might decrease bladder cancer recurrence and greatly benefit bladder cancer survivors.

## Conclusions

In conclusion, we confirm decreased proliferation of bladder cancer cells by treatment with HDAC inhibitors and show increased expression of TSP1 in bladder cancer by this class of drug. This is a novel mechanism for bladder cancer control which can be exploited in future clinical trials.

## Abbreviations

TSP1: thrombospondin-1; HDACi: histone deacetylase inhibitors; PBS: phosphate buffered saline; SAHA: vorinostat.

## Competing interests

The authors declare that they have no competing interests.

## Author’s contributions

TKB and JER performed the experiments. All authors contributed to study design and the conceptual framework TKB, JER drafted the manuscript and all authors reviewed the final version.

## Pre-publication history

The pre-publication history for this paper can be accessed here:

http://www.biomedcentral.com/1471-2490/12/21/prepub
